# Random plasma glucose predicts the diagnosis of diabetes

**DOI:** 10.1371/journal.pone.0219964

**Published:** 2019-07-19

**Authors:** Mary K. Rhee, Yuk-Lam Ho, Sridharan Raghavan, Jason L. Vassy, Kelly Cho, David Gagnon, Lisa R. Staimez, Christopher N. Ford, Peter W. F. Wilson, Lawrence S. Phillips

**Affiliations:** 1 Atlanta VA Health Care System, Decatur, Georgia, United States of America; 2 Department of Medicine, Division of Endocrinology and Metabolism, Emory University School of Medicine, Atlanta, Georgia, United States of America; 3 MAVERIC VA Boston Healthcare System, Boston, Massachusetts, United States of America; 4 VA Eastern Colorado Healthcare System, Aurora, Colorado, United States of America; 5 Department of Medicine, Division of Hospital Medicine, University of Colorado School of Medicine, Aurora, Colorado, United States of America; 6 Department of Medicine, Division of General Internal Medicine, Brigham and Women’s Hospital, Boston, Massachusetts, United States of America; 7 Department of Medicine, Harvard Medical School, Boston, Massachusetts, United States of America; 8 Department of Medicine, Department of General Aging, Brigham and Women’s Hospital, Boston, Massachusetts, United States of America; 9 Department of Biostatistics, Boston University School of Public Health, Boston, Massachusetts, United States of America; 10 Hubert Department of Global Health, Rollins School of Public Health, Emory University, Atlanta, Georgia, United States of America; 11 Department of Medicine, Division of Cardiology, Emory University School of Medicine, Atlanta, Georgia, United States of America; Shanghai Diabetes Institute, CHINA

## Abstract

**Aims/Hypothesis:**

Early recognition of those at high risk for diabetes as well as diabetes itself can permit preventive management, but many Americans with diabetes are undiagnosed. We sought to determine whether routinely available outpatient random plasma glucose (RPG) would be useful to facilitate the diagnosis of diabetes.

**Methods:**

Retrospective cohort study of 942,446 U.S. Veterans without diagnosed diabetes, ≥3 RPG in a baseline year, and ≥1 primary care visit/year during 5-year follow-up. The primary outcome was incident diabetes (defined by diagnostic codes and outpatient prescription of a diabetes drug).

**Results:**

Over 5 years, 94,599 were diagnosed with diabetes [DIAB] while 847,847 were not [NONDIAB]. Baseline demographics of DIAB and NONDIAB were clinically similar, except DIAB had higher BMI (32 vs. 28 kg/m^2^) and RPG (150 vs. 107 mg/dl), and were more likely to have Black race (18% vs. 15%), all p<0.001. ROC area for prediction of DIAB diagnosis within 1 year by demographic factors was 0.701, and 0.708 with addition of SBP, non-HDL cholesterol, and smoking. These were significantly less than that for prediction by baseline RPG alone (≥2 RPGs at/above a given level, ROC 0.878, p<0.001), which improved slightly when other factors were added (ROC 0.900, p<0.001). Having ≥2 RPGs ≥115 mg/dl had specificity 77% and sensitivity 87%, and ≥2 RPGs ≥130 mg/dl had specificity 93% and sensitivity 59%. For predicting diagnosis within 3 and 5 years by RPG alone, ROC was reduced but remained substantial (ROC 0.839 and 0.803, respectively).

**Conclusions:**

RPG levels below the diabetes “diagnostic” range (≥200 mg/dl) provide good discrimination for follow-up diagnosis. Use of such levels–obtained opportunistically, during outpatient visits–could signal the need for further testing, allow preventive intervention in high risk individuals before onset of disease, and lead to earlier identification of diabetes.

## Introduction

Diabetes mellitus–largely type 2 diabetes–is a major public health problem [1]. Diabetes is the major cause of kidney failure, blindness, and nontraumatic leg amputations in adults in the U.S., and a leading cause of stroke and heart disease. The diagnosis of diabetes triggers initiation of care, and many newly-diagnosed patients will have sufficient life expectancy and lack of comorbidities to be candidates for good metabolic control, aimed at preventing or delaying the development of complications which increase morbidity, mortality, and costs. However, over 7 million Americans remain undiagnosed [1].

Moreover, since the diagnosis can be delayed, patients may have characteristic microvascular diabetes complications when they are first diagnosed [2], particularly in populations enriched with racial/ethnic minorities. Based on extrapolations from the prevalence of diabetic retinopathy, older work suggested that the onset of type 2 diabetes might be as much as 9–12 years before clinical diagnosis in the U.S. and Australia [3], although more recent evaluations found a delay of 6 years in Italy [4] and 2.4 years even with a *de facto* screening program in the Atherosclerosis Risk in Communities (ARIC) study in the U.S. [5].

Systematic screening could permit detection earlier in the natural history [6], when use of lifestyle change strategies and/or medications could help prevent or delay progression from prediabetes to diabetes [7], and the development of diabetes complications [8]. Although screening of high-risk patients might be cost-effective [9], many healthcare systems have not yet implemented widespread screening. Accordingly, we need convenient, inexpensive ways to identify prediabetes or undiagnosed diabetes. We investigated whether levels of outpatient random plasma glucose (RPG), frequently included in laboratory tests obtained during routine visits, although below “diagnostic” thresholds [≥200 mg/dl [10]], predict subsequent diabetes diagnosis, and thus could be used to identify patients who might benefit from more definitive assessment and preventive management.

## Research design and methods

### Study design

We conducted a retrospective analysis of the association of outpatient random plasma glucose (RPG) levels with the subsequent clinical diagnosis of diabetes. Study subjects were Veterans with data in the Veterans Administration (VA) Informatics and Computing Infrastructure (VINCI) Corporate Data Warehouse (CDW). The CDW contains data on all Veterans receiving care in Veterans Health Administration (VHA) facilities nationwide, including demographics, vital signs, laboratory tests, diagnoses, procedures, and prescriptions. If Veterans were ≥65 years old, their data were complemented with data from the Centers for Medicare and Medicaid Services (CMS) databases.

We selected Veterans who (a) did not have a diabetes diagnosis during a baseline year (365 days) in 2002–2007; (b) had 3 or more measurements of random plasma/serum glucose (RPG) during the baseline year, along with assessments of other demographic variables and cardiovascular disease (CVD) risk factors; and (c) had subsequent continuity of primary care [≥1 primary care provider (PCP) visit per year for 5 years immediately following the baseline year]. Diabetes was defined by both (a) either ≥1 use of the International Classification of Diseases, Ninth Revision (ICD-9) code 250.xx at a PCP visit, or ≥2 uses of the code in any setting, and (b) an outpatient prescription of a diabetes drug (based on use of VHA national drug codes); inclusion of this criterion increased specificity compared to previous validations [11]. There were 8,401,688 Veterans who had at least one VHA visit in 2002–2007 and other demographic variables; among this group, 6,578,661 had at least one PCP visit, of whom 5,052,534 had at least one outpatient RPG in that time period; 2,557,751 had at least three RPG levels within one year (365 days); 1,457,036 also did not have diabetes at baseline; 972,132 had continuity of care; and 942,446 did not have any RPG ≥200 mg/dl (S1 Fig and S1 Table). Patients with 3 or more RPG levels in the baseline period were not clinically different in age, race, sex, BMI or comorbidity score from those with more than 1 or more than 2 RPG levels, but had slightly higher RPG levels (106 vs 103 and 104 mg/dl, respectively) and were more likely to have preexisting CVD (38% vs. 33% and 35%, respectively) (S2 Table). Within the following 5 years, 94,599 were diagnosed with diabetes, while 847,847 were not. The Emory University and VA Boston Institutional Review Boards and the Atlanta VA Medical Center and Boston VA Research and Development Committees approved this study and granted a HIPAA waiver obviating the need for subject consent.

### Analysis

Race was characterized using CDW data as white, black, and other, and ethnicity as Hispanic/Latino and not Hispanic/Latino. All laboratory determinations were performed by the clinical chemistry laboratories at the VHA facilities where subjects were receiving care; inpatient determinations and outpatient determinations on the days of hospital admission and discharge were excluded. CVD at baseline was assessed according to use of ICD-9 diagnosis and procedure codes [12]. CVD risk factors included body mass index (BMI, kg/m^2^), systolic blood pressure (SBP, mmHg), and non-high density lipoprotein cholesterol levels (non-HDL cholesterol, mg/dl); if more than one measurement was available, values closest to patient visits were taken. Smoking was classified by updating a previously validated approach [13]. Patients with missing data were not included in the analysis.

Chi-square tests were used for categorical variables; ANOVA and two-tailed t-tests to evaluate means of continuous variables; and Kruskal-Wallis and Wilcoxon tests to evaluate median values of outcomes which were not normally distributed. C-statistic (receiver operating characteristic, ROC) analysis was used to evaluate the accuracy of prediction of the clinical diagnosis of diabetes according to RPG values during a baseline year, adjusting for potential confounders including demographic factors and cardiovascular risk factors, along with integrated discrimination improvement (IDI) as a measure of improved discrimination. To account for the possibility that early morning RPG values might reflect fasting glucose levels–which could provide diagnostic results for diabetes and prediabetes with glucose levels ≥126 and 100 mg/dl, respectively–predictive performance with RPG measured at different times of the day was also evaluated by using ROC analysis. Sensitivities and specificities for predicting incident diabetes at 1, 3, and 5 years were calculated for different RPG thresholds.

All statistical analyses utilized SAS Enterprise Guide 7.1 (Cary, NC) and STATA MP14 (College Station, TX).

## Results

### Baseline patient characteristics

Patient characteristics are shown in Table 1. As expected for a Veteran population, the subjects were predominantly male and white. Ten percent developed diabetes by the end of the 5-year study period, with an annual incidence of meeting criteria for diabetes (“incident diabetes”) of 4.3% in the first year, followed by 1.7%, 1.6%, 1.5%, and 1.4% in each subsequent year. Minority race/ethnicity, younger age, higher BMI, and higher non-HDL cholesterol were more common in those who developed diabetes, compared to those who remained diabetes-free (all p<0.001). The median RPG level for the baseline year and 1 year prior was 101 mg/dl (90^th^ percentile 114, 95^th^ percentile 120 mg/dl) among the 847,847 patients who remained nondiabetic during the five year study period, and 112 mg/dl (90^th^ percentile 135, 95^th^ percentile 145 mg/dl) among those who developed incident diabetes (S3 Table).

**Table 1:** Patient characteristics at baseline.

	All	Diabetes Status after 5 years of follow-up		p-value
		Non-Diabetic	Diabetic	
**n**	942,446	847,847	94,599	
**Age (years)**	63.4 ± 12.4	63.7 ± 12.6	61.0 ± 10.1	<0.001
**Male sex**	96.1%	96.0%	96.8%	<0.001
**Race**				
White	82.7%	83.1%	79.7%	<0.001
Black	14.9%	14.6%	17.5%	<0.001
Other	2.4%	2.3%	2.8%	<0.001
**Ethnicity**				
NonHispanic/Unknown	95.1%	95.2%	94.4%	<0.001
Hispanic/Latino	4.9%	4.8%	5.6%	
**BMI (kg/m**^**2**^**)**	28.9 ± 5.4	28.46 ± 5.2	32.42 ± 5.9	<0.001
**Systolic BP (mmHg)**	134.6 ± 18.6	134.5 ± 18.6	135.8 ± 17.8	<0.001
**Non-HDL Chol (mg/dl)**	141.5 ± 39.8	140.9 ± 39.5	146.9 ± 41.2	<0.001
**RPG glucose (mg/dl)**	108.8 ± 22.1	106.3 ± 20.2	132.0 ± 24.8	<0.001
**Smoking (past/current)**	81.3%	81.2%	82.3%	<0.001
**CVD, preexisting**	37.5%	37.4%	38.4%	<0.001
**Average baseline year**	2004	2004	2005	<0.001

### Prediction of diabetes diagnosis within 1, 3, and 5 years

Receiver operating characteristic (ROC) analysis permits an assessment of predictive accuracy which is both independent of any particular diagnostic cutoff (the area under the ROC curve [AUC] is a measure of the accuracy of all possible cutoffs), and independent of the probability of the outcome of interest in any group of test subjects (for example, in those who have higher or lower age, or BMI–see below). At one, three, and five year followup, the ROCs for using the average RPG and for at least 2 RPGs at/above any specified cutoff were generally comparable, but both were greater than that for using maximum RPG in predicting diabetes (all p<0.0001). S2 Fig shows the ROC curves for the three approaches for assessing RPG during the baseline year, to predict a diagnosis of diabetes during the following year. Given its practical utility in the clinical setting compared to average RPG, having at least 2 RPGs at/above a threshold was used for all subsequent analyses of RPG as a predictor of diabetes.

**Fig 1:**
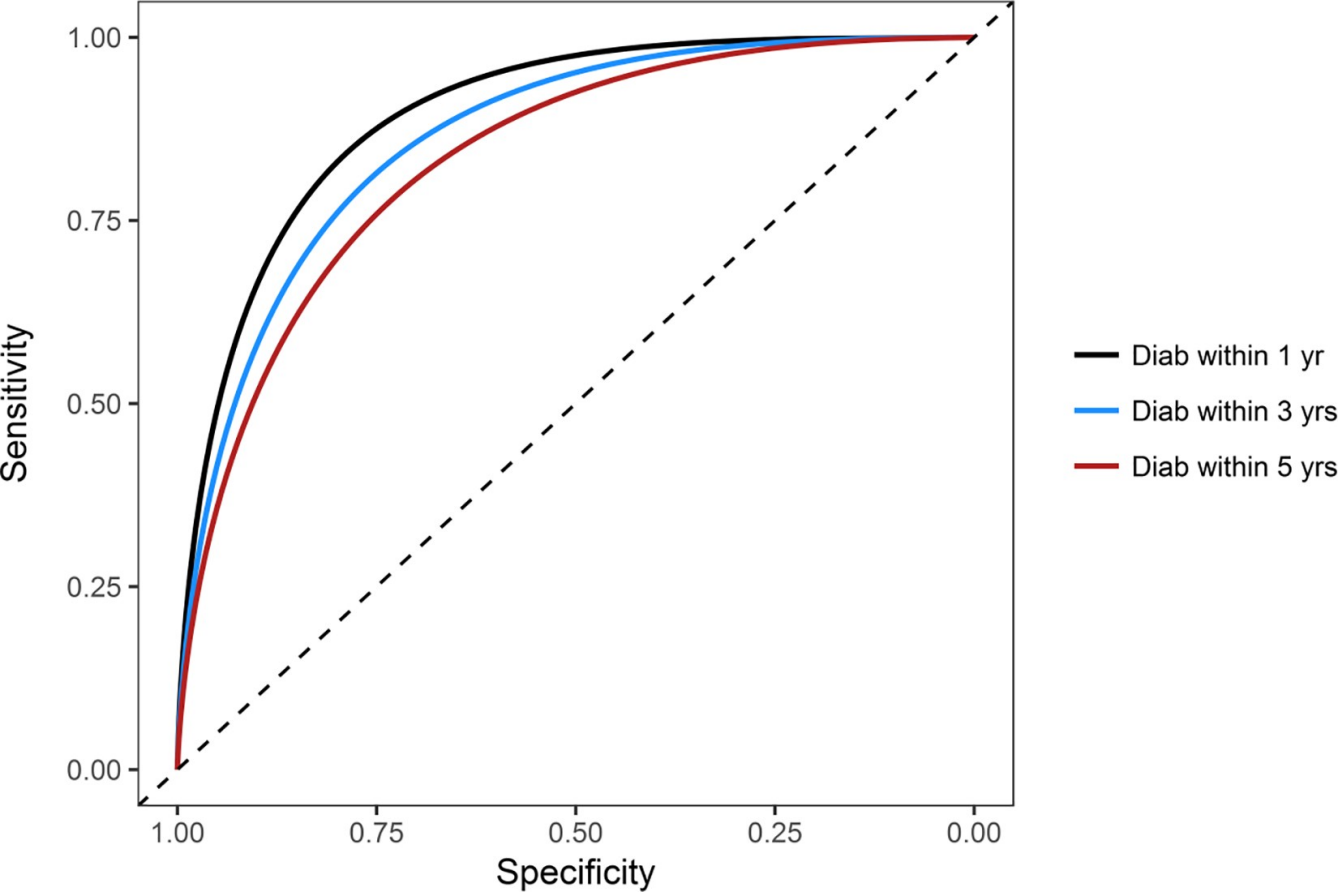
ROC curves for at least 2 RPGs ≥ a cutoff to predict a diagnosis of diabetes within 1, 3, and 5 years. p<0.0001 for ROC AUC for 1 year vs 3 years, and for 3 years vs 5 years.

The ROC area under the curve for prediction of meeting criteria for a diagnosis of diabetes (ROC) within one year after the baseline year was 0.701 based on demographic factors (age, sex, race/ethnicity and BMI), and increased modestly to 0.708 with further inclusion of CVD risk factors (SBP, non-HDL cholesterol, and smoking status), p<0.0001 (Table 2). However, with further addition of RPG (using the approach of having at least 2 RPG measures greater than or equal to any specified cutoff), there was a marked improvement in ROC AUC (to 0.900, p<0.0001), with a similar highly significant increase in integrated discrimination improvement (Table 2). In contrast, the ROC AUC for diagnosis within one year after the baseline year was 0.878 based on RPG alone (at least 2 RPGs greater than or equal to a cutoff), and was increased to 0.900 with addition of demographic and CVD risk factors (p<0.0001). Similar findings were observed when each of the above analyses was stratified by sex (S4 Table). We analyzed the ROC for a diagnosis within one, three, and five years by different approaches to assessing RPG during the baseline year: average RPG, at least 2 RPGs at/above any specified cutoff, and maximum RPG (Table 2). With each approach to expressing RPG during the baseline year, the ROC for diagnosis within one year was greater than that for a diagnosis within three years, and greater for prediction of a diagnosis within three compared to within five years (all p<0.0001). Fig 1 shows the ROC curves for at least 2 RPGs at/above any specified cutoff to predict a diagnosis of diabetes within one, three and five years.

**Table 2:** ROCs and integrated discrimination improvement (IDI) for prediction of diabetes at years 1, 3, and 5.

	Models	Year 1	Year 3	Year 5
**ROC AUC (95**^**th**^**% CI)**	Model 1: Demographic factors ^a^	0.701 (0.699, 0.704)	0.703 (0.701, 0.705)	0.702 (0.700, 0.704)
	Model 2: Demographic + CVD factors ^b^	0.708 (0.705, 0.710)	0.705 (0.703, 0.707)	0.703 (0.701, 0.705)
	Model 3: RPG (maximum)	0.856 (0.855, 0.858)	0.822 (0.820, 0.823)	0.791 (0.790, 0.793)
	Model 4: RPG (average)	0.877 (0.876, 0.879)	0.836 (0.834, 0.837)	0.799 (0.798, 0.801)
	Model 5: RPG (≥2 measures at/above threshold)	0.878 (0.876, 0.880)	0.839 (0.837, 0.840)	0.803 (0.802, 0.805)
	Model 6: Demographic + CVD + RPG (maximum)	0.882 (0.881, 0.884)	0.855 (0.854, 0.857)	0.832 (0.831, 0.833)
	Model 7: Demographic + CVD + RPG (average)	0.899 (0.898, 0.901)	0.869 (0.868, 0.871)	0.843 (0.842, 0.845)
	Model 8: Demographic + CVD + RPG (≥2 at/above threshold)	0.900 (0.899, 0.902)	0.872 (0.870, 0.873)	0.847 (0.846, 0.848)
**IDI** ^c^	Model 1: Demographic factors ^a^	reference	reference	reference
	Model 2: Demographic + CVD factors ^b^	0.0008	0.0011	0.0014
	Model 3: Demographic + CVD + RPG (≥2 at/above threshold)	0.1872	0.2456	0.2615

n = 941,561, including only subjects with all CVD risk factors

^a^ Demographic factors (age, sex, BMI, race, ethnicity)

^**b**^ CVD factors (systolic blood pressure, non-HDL-cholesterol, smoking)

^c^ p<0.0001 for all IDI analyses comparing prediction performances using model 1 as reference within each year

### Sensitivity, specificity, and positive and negative predictive value

Table 3 shows sensitivity, specificity, and positive and negative predictive value for different cutoffs for at least 2 RPGs at/above any specified cutoff to predict a diagnosis of diabetes within one, three, and five years. For a diagnosis within three years, a cutoff ≥115 mg/dl had almost equal sensitivity and specificity, and a cutoff ≥ 130 mg/dl had nearly 95% specificity. Findings were similar when stratified by sex, except females had a sensitivity and specificity of 80% and 78%, respectively, at a slightly lower RPG threshold of ≥110 mg/dl compared to males who had a similar sensitivity and specificity at a RPG threshold of ≥115 mg/dl (S5 Table). There were 245,740 patients (26.1% of our study subjects) who had at least 2 RPGs ≥115 mg/dl in the baseline year. The positive predictive value was nearly 40% when the RPG cutoff was ≥130 mg/dl, and over 20% when the cutoff was ≥115 mg/dl; nearly a quarter of those with at least 2 RPGs ≥115 mg/dl were diagnosed with diabetes within 3 years. When using a RPG cutoff ≥130 mg/dl, the specificity and PPV are similar to that observed with the fasting plasma glucose ≥126 mg/dl [14]. The negative predictive values (NPV) of RPGs <110 mg/dl for *not* being diagnosed with diabetes within one, three, and five years are shown in S6 Table. For *not* being diagnosed with diabetes within three years, the NPV of 1, 2, and 3 RPGs <110 mg/dl were 96.0%, 98.5%, and 99.0%, respectively.

**Table 3:** Sensitivity, sensitivity, specificity, and positive and negative predictive values for different cutoffs for at least 2 RPGs at/above a cutoff to predict a diagnosis of diabetes within 1, 3, and 5 years.

≥2 measures at/above RPG threshold (mg/dl)	n	%	Year 1				Year 3				Year 5			
			SENS ^a^	SPEC ^b^	PPV^c^	NPV^d^	SENS ^a^	SPEC ^b^	PPV ^c^	NPV^d^	SENS ^a^	SPEC ^b^	PPV ^c^	NPV^d^
110	349,357	37.1	92.0%	65.4%	10.6%	99.5%	86.9%	66.9%	17.4%	98.5%	81.7%	67.9%	22.1%	97.1%
115	245,740	26.1	86.5%	76.6%	14.2%	99.2%	78.6%	78.1%	22.3%	97.9%	71.5%	79.0%	27.5%	96.1%
120	172,535	18.3	79.5%	84.4%	18.6%	98.9%	68.9%	85.7%	27.9%	97.2%	60.6%	86.4%	33.2%	95.2%
130	85,346	9.1	58.7%	93.2%	27.7%	98.1%	46.4%	93.9%	37.9%	95.6%	38.7%	94.3%	42.9%	93.2%
140	44,059	4.7	38.3%	96.8%	35.0%	97.2%	29.0%	97.3%	46.0%	94.5%	23.7%	97.4%	50.8%	92.0%
150	24,447	2.6	25.5%	98.4%	41.9%	96.7%	18.9%	98.7%	54.0%	93.8%	15.1%	98.8%	58.5%	91.3%

^a^ SENS = sensitivity

^b^ SPEC = specificity

^c^ PPV = positive predictive value

^d^ NPV = negative predictive value

### Predictive performance with RPG measured at different times of the day

RPG was most predictive of diabetes within one, three and five years when samples were collected before 9 AM, somewhat less predictive with collection later in the morning and early afternoon, but then more predictive when sample were collected after 3 PM (S7 Table).

### Predictive performance in subjects with different risk of developing diabetes

Although factors such as age, sex, race, ethnicity, BMI, smoking, SBP and non-HDL cholesterol increased the likelihood of developing diabetes, the predictive performance of RPG was similar in those at higher and lower risk; the ROC AUC ranged from 0.84–0.88 for each subgroup, as shown in Table 4. However, the risk for incident diabetes was much higher with even modest elevation in RPG–odds ratio (OR) 5.4 for RPG 110–119 mg/dl, and OR 45.3 for RPG 130–139 (all p<0.0001).

**Table 4:** Risk of incident diabetes within 1, 3, and 5 years ^a^.

Risk factors		ROC area (95^th^ % CI) ^b^			OR (95^th^% CI) of incident diabetes		
		Year 1	Year 3	Year 5	Year 1 ^c^	Year 3 ^c^	Year 5 ^c^
**Age,** yrs	<40	0.87 (0.85, 0.89)	0.82 (0.80, 0.83)	0.77 (0.76, 0.78)	1.00	1.00	1.00
	40–55	0.88 (0.87, 0.88)	0.83 (0.83, 0.83)	0.79 (0.79, 0.79)	2.24 (2.06, 2.43)	2.04 (1.93, 2.17)	1.94 (1.85, 2.03)
	>55	0.87 (0.87, 0.88)	0.84 (0.84, 0.84)	0.81 (0.80, 0.81)	2.24 (2.07, 2.43)	1.90 (1.79, 2.01)	1.64 (1.56, 1.72)
**Sex**	Female	0.87 (0.86, 0.88)	0.83 (0.82, 0.84)	0.79 (0.78, 0.79)	1.00	1.00	1.00
	Male	0.88 (0.87, 0.88)	0.84 (0.83, 0.84)	0.80 (0.80, 0.80)	1.43 (1.35, 1.52)	1.32 (1.27, 1.38)	1.27 (1.22, 1.32)
**Race**	White	0.88 (0.88, 0.88)	0.84 (0.84, 0.85)	0.81 (0.81, 0.81)	1.00	1.00	1.00
	Black	0.84 (0.83, 0.85)	0.80 (0.79, 0.80)	0.76 (0.76, 0.77)	1.14 (1.11, 1.17)	1.20 (1.18, 1.23)	1.25 (1.23, 1.27)
	Other	0.87 (0.86, 0.88)	0.84 (0.83, 0.85)	0.80 (0.79, 0.81)	1.28 (1.21, 1.36)	1.26 (1.21, 1.33)	1.28 (1.23, 1.34)
**Ethnicity**	NonHispanic/Unk	0.88 (0.87, 0.88)	0.84 (0.83, 0.84)	0.80 (0.80, 0.80)	1.00	1.00	1.00
	Hispanic/Latino	0.87 (0.86, 0.88)	0.83 (0.82, 0.84)	0.80 (0.79, 0.80)	1.17 (1.12, 1.22)	1.18 (1.14, 1.22)	1.17 (1.14, 1.21)
**BMI,** kg/m^2^	<25	0.85 (0.85, 0.86)	0.81 (0.80, 0.82)	0.77 (0.77, 0.78)	1.00	1.00	1.00
	25–29	0.88 (0.88, 0.88)	0.84 (0.83, 0.84)	0.80 (0.80, 0.80)	2.70 (2.59, 2.83)	2.55 (2.47, 2.64)	2.41 (2.34, 2.47)
	≥30	0.86 (0.86, 0.86)	0.82 (0.82, 0.83)	0.79 (0.79, 0.79)	7.64 (7.32, 7.97)	7.09 (6.87, 7.31)	6.59 (6.43, 6.76)
**Smoking**	Never	0.88 (0.87, 0.88)	0.84 (0.84, 0.85)	0.81 (0.80, 0.81)	1.00	1.00	1.00
	Past/current	0.87 (0.87, 0.88)	0.83 (0.83, 0.84)	0.80 (0.80, 0.80)	1.07 (1.04, 1.1)	1.08 (1.05, 1.1)	1.08 (1.06, 1.1)
**Systolic blood pressure,** mmHg	<140	0.87 (0.87, 0.88)	0.83 (0.83, 0.84)	0.80 (0.80, 0.80)	1.00	1.00	1.00
	≥140	0.88 (0.88, 0.88)	0.84 (0.84, 0.84)	0.80 (0.80, 0.81)	1.14 (1.12, 1.17)	1.11 (1.1, 1.13)	1.09 (1.08, 1.11)
**Non-HDL cholesterol,** mg/dl	<100	0.84 (0.84, 0.85)	0.81 (0.80, 0.81)	0.78 (0.77, 0.78)	1.00	1.00	1.00
	≥100	0.88 (0.88, 0.88)	0.84 (0.84, 0.84)	0.80 (0.80, 0.81)	1.55 (1.51, 1.6)	1.47 (1.44, 1.51)	1.47 (1.44, 1.5)
**Random plasma glucose,** mg/dl	<110				1.00	1.00	1.00
	110–119				5.35 (5.11, 5.59)	4.85 (4.72, 4.98)	4.24 (4.15, 4.33)
	120–129				19.54 (18.75, 20.36)	14.05 (13.68, 14.43)	10.36 (10.13, 10.58)
	130–139				45.3 (43.43, 47.26)	26.44 (25.67, 27.24)	17.49 (17.06, 17.94)
	≥140				98.67 (94.82, 102.67)	54.22 (52.73, 55.75)	34.32 (33.51, 35.16)

^**a**^ n = 941,561, including only subjects with all CVD risk factors

^**b**^ ROC area for at least 2 RPGs greater than or equal to any specified cutoff

^**c**^ p<0.0001 for all comparisons to the reference group in each category

## Discussion

In close to one million Veterans who had at least three measurements of outpatient random plasma glucose (RPG) during a baseline year in 2002–2007, continuity of primary care for the following five years, and no known diagnosis of diabetes, RPG levels at baseline were strongly predictive of meeting criteria for the diagnosis of diabetes during follow-up (use of diabetes diagnostic codes, and outpatient prescription of a diabetes drug). A diagnosis of diabetes during follow-up was also predicted by baseline demographics (age, sex, race/ethnicity, and BMI) and cardiovascular disease (CVD) risk factors (SBP, non-HDL cholesterol levels, and smoking history). However, the ROC by such factors within one, three, and five years of follow-up were all significantly less than the ROC for prediction by baseline RPG alone, which was increased only modestly by combination with demographics and CVD risk factors. Moreover, the ROC was similar in groups of subjects with different risks of developing diabetes–such as higher or lower BMI, or age. Although RPG levels ≥200 mg/dl (in combination with symptoms) are considered diagnostic [10], considerably lower levels appeared to confer substantial risk: having at least 2 RPGs ≥115 mg/dl predicted incident diabetes within one year with specificity 77% and sensitivity 87%, and having at least 2 RPGs ≥130 mg/dl had specificity 93% and sensitivity 59%. Accordingly, our findings indicate that strong consideration should be given to follow-up diagnostic testing [10] in patients who within one year, have two outpatient RPGs ≥115 mg/dl and especially those with two RPGs ≥130 mg/dl. Use of the ≥115 mg/dl RPG cutoff could also be justified since it represents the 90^th^ percentile of the median baseline RPG level in the group that was nondiabetic at baseline and remained nondiabetic for the next five years.

There has been little previous examination of RPG as a predictor of incident diabetes, and none that we are aware of in the context of a health care system as opposed to a research study. In the Study of Health in Pomerania (SHIP), with a relatively homogeneous population, RPG at baseline predicted incident diabetes within 5 years with an ROC of 0.72 [15]. This is lower than the 5-year ROC in the present study with at least 2 RPGs at or above a given level (0.803 [Table 2]), despite our evaluation of a more diverse population, a difference potentially attributable both to the use of more specific diagnostic criteria in the present study vs. unvalidated self-report in the SHIP study, and the use of multiple RPG measurements in the present study vs. a single RPG in the SHIP study. Another study analyzing the U.S. National Health and Nutrition Examination Survey (NHANES) 2007–2012 data to determine the potential utility of a random glucose level to identify individuals in need of diabetes testing, showed that use of a pre-selected RPG threshold of ≥100 mg/dl was both highly sensitive and specific, and had higher ROC for identifying undiagnosed diabetes compared to both the American Diabetes Association (ADA) and U.S. Preventive Services Task Force (USPSTF) screening strategies [16]. However, this analysis was limited by its use of HbA1c alone for determination of diabetes diagnosis, which increases the risk for misclassification, and only evaluated the utility of RPG in the identification of prevalent diabetes, rather than prediction of future diabetes diagnosis.

Our findings are also consistent with previous studies comparing fasting plasma glucose (FPG) with nonglycemic factors for predicting risk of incident diabetes; adding FPG significantly improved prediction in Dutch, Korean, Japanese, and U.S. populations [17–20], and glycemic markers such as FPG and HbA1c were generally superior to nonglycemic factors [21]. However, the 2-hour plasma glucose level during an oral glucose tolerance test (OGTT) may be less predictive than a model combining FPG with nonglycemic factors, and may add little when combined with such a model [22]. At this time, it appears that addition of genetic or metabolomic markers provides little improvement in diabetes risk prediction beyond that of conventional risk factors [23, 24]. These studies were also generally consistent with our observation that while prediction of diabetes risk by RPG alone was substantially better than demographic and CVD risk factors in combination, addition of the nonglycemic factors to RPG did improve prediction of risk (Table 2).

In the present study, the ROC for predicting incident diabetes within five years by at least 2 RPGs at/above a given level (0.803 unadjusted and 0.847 adjusted [Table 2]), was similar to that for prediction of diabetes at 7–8 years by a single 1-hour OGTT glucose in the San Antonio Heart Study [0.84 [25]] and the Botnia Study [0.80 [26]]. Such comparability may be due in part to the shorter time period in the present study (5 years vs. 7–8 years), since we found that ROC areas were higher with shorter time periods (Table 2). As such, it seems likely that RPG at baseline is more representative of proximal diabetes risk compared to risk at three and five years later. For example, an RPG level at baseline may fall within the “normal” range, then increase over time in individuals at future risk, i.e., those beginning to develop higher glucose levels more than one or three years later. However, a single RPG was less accurate than a single plasma glucose 1 hour after a 50g oral glucose challenge (GCTplasma) or a single 1-hour OGTT plasma glucose for identification of prevalent diabetes in the Screening for Impaired Glucose Tolerance (SIGT) study, with ROC areas of 0.83 vs. 0.90 and 0.93, respectively [p = ns and p<0.05, respectively, [27]].

While our study was focused on 942,446 individuals who had at least 3 outpatient RPG measurements during a baseline year, even in a larger group of 1,410,505 individuals who had at least 2 outpatient RPGs during a baseline year, use of RPG levels (≥2 values above a threshold) was only slightly less strong in predicting incident diabetes, with ROC AUCs of 0.870, 0.816, and 0.775 for incident diabetes during 1, 3, and 5 years, respectively (all p<0.001). Moreover, among Veterans without a known diagnosis of diabetes at baseline and with at least one PCP visit, 60% had at least two RPGs, suggesting that a large proportion of the at-risk population could easily be screened using this opportunistic approach (S1 Table).

The strengths of our study include large size, diversity of the subjects, and a national sample. Limitations include restriction to Veterans, and a predominance of male sex and white race, although there were a large number of women and minority subjects, and the ROC for prediction of diabetes was similar in subjects of different race, ethnicity, and sex (Table 4). Moreover, the utility of RPG as a screening test for diabetes or prediabetes is limited by its potential variability dependent on factors such as nutrition status (i.e., temporal relationship to food ingestion, the quantity and macronutrient content of the food/drink ingested), stress, physical activity level, medications affecting glucose homeostasis (i.e., glucocorticoids), acute illness, and diseases of the pancreas, liver or kidney. While RPG measures are not standardized compared to FPGs or OGTTs, the tests are opportunistic (often performed when patients come for outpatient visits), and thus relatively inexpensive and convenient, without the requirement for a prior fast. We were unable to assess the impact of time since the prior meal on the utility of RPG levels, as delineating fasting vs. nonfasting glucose levels cannot be done accurately in an administrative database. In a prior study, shorter time periods after the previous meal appeared to improve the accuracy of identification of prevalent diabetes by random capillary glucose levels [28], but did not affect the accuracy of plasma glucose after an oral glucose challenge test (GCTplasma) [27]. However, for predicting diabetes within 3 years, ROC areas appeared to be modestly lower, but still significantly predictive, during the middle of the day, compared to collections in the morning and late afternoon (0.78 between 1–3 pm vs 0.87 before 9 am and 0.81 at 3–5 pm). Since regulation of glucose homeostasis is dependent on adequate insulin secretion from the beta cells of the pancreas, insulin sensitivity of insulin-dependent tissues, particularly the liver and muscle, adequate metabolism of insulin, and regulation of counterregulatory hormones, such as glucagon, medical conditions affecting any of these factors may alter RPG levels and its utility as a prediction of diabetes risk. We did not determine the impact of comorbid conditions, such as pancreatitis, history of pancreatectomy, chronic liver disease, or chronic kidney disease, or whether some patients were receiving glucocorticoids or other medications which can influence glucose levels; inclusion of such variability in our study population would have tended to reduce predictive accuracy. Although meeting criteria for a diagnosis of diabetes was based on previously validated criteria [11], and we attempted to increase specificity by requiring the outpatient prescription of a diabetes medication, the potential use of RPG levels to predict diabetes risk needs further validation in other settings and other populations, and ideally with confirmation by definitive diagnostic tests [10]. Future analyses are also needed to investigate how the two-level RPG strategy compares with HbA1c testing for screening; in screening for prevalent diabetes or prediabetes, a single RPG measurement was slightly but not significantly superior to HbA1c screening in our previous studies [29, 30].

Our findings suggest that outpatients who have at least 2 RPG levels of 115 mg/dl or higher should be considered for further evaluation to determine if they have diabetes or prediabetes; diagnostic testing may be particularly cost-effective if patients have additional risk factors [9]. Higher cutoffs may be more cost-effective for detecting diabetes alone, and lower cutoffs for detecting diabetes or prediabetes [31], although whether more sensitive or more specific cutoffs will be more cost-effective depends on the projected costs of testing and care. The selection of a cutoff can also be guided in part by glucose levels relative to the “normal range”. While the normal range of RPG has not been established, in the subgroup of our cohort which did not develop diabetes during the 5-year follow-up period [NONDIAB], only 10% had a median baseline RPG level ≥115 mg/dl, and only 5% had a median RPG level ≥120 mg/dl, consistent with the high specificity of these RPG threshold levels for the diagnosis of diabetes during follow-up. Use of opportunistically available RPG levels can therefore help to identify a subgroup of individuals at higher risk for developing diabetes, and then to implement a stepwise approach of additional screening/testing using any of the three standard diagnostic tests (fasting plasma glucose, OGTT, A1c) as recommended by the ADA.

In conclusion, random plasma glucose levels well below the conventional “diagnostic” range appear to provide good discrimination for follow-up diagnosis of diabetes. Use of such levels–obtained opportunistically, during outpatient visits–to signal the need for definitive testing might lead to earlier identification, and permit initiation of preventive management, but would need confirmation in other cohorts.

## Supporting information

S1 Table Number of RPG measurements in baseline year in U.S. veterans with a PCP visit, stratified by baseline diabetes status. (PDF)

S2 Table Patient characteristics by number of available RPGs in baseline year. (PDF)

S3 Table Percentile distribution of median RPG levels at baseline and 1 year prior, stratified by diabetes status at end of followup period. (PDF)

S4 Table ROCs and integrated discrimination improvement (IDI) for prediction of diabetes at years 1, 3, and 5, stratified by sex. (PDF)

S5 Table Sensitivity, sensitivity, specificity, and positive and negative predictive values for different cutoffs for at least 2 RPGs at/above a cutoff to predict a diagnosis of diabetes within 1, 3, and 5 years, stratified by sex. (PDF)

S6 Table Negative predictive values of RPG values <110 mg/dl for *not* being diagnosed with diabetes within 1, 3, and 5 years. (PDF)

S7 Table ROCs for prediction of diabetes by collection time of RPG. Total n = 653,180, only subjects having 2 out of 3 glucose measurements collected at the same time of day.
(PDF)

S1 Fig CONSORT diagram. (TIF)

S2 Fig ROC curves for 3 approaches for assessing RPG during the baseline year, to predict a diagnosis of diabetes during the following year. p<0.0001 for ROC AUC for average RPG vs maximum RPG, and for ≥2 RPGs at/above a cutoff vs maximum RPG; p = NS for ROC AUC for average RPG vs 2 RPGs ≥ a cutoff.
(TIF)
