# Supplementary material for: Random plasma glucose predicts the diagnosis of diabetes
Source: PLoS One. 2019 Jul 19;14(7):e0219964. doi: 10.1371/journal.pone.0219964 (PMC6641200; doi:10.1371/journal.pone.0219964)
Supplement: S1 Table — (PDF) [file pone.0219964.s001.pdf]

S1 Table: Number of RPG measurements in baseline year in Veterans with a PCP visit, stratified by baseline diabetes status

# RPG measures in baseline year	Diabetes status at baseline	
	**Diabetes** n=6,578,661	**No Diabetes** n=4,957,674
**0**	1,526,127 (23.1%)	1,055,229 (21.2%)
**1**	1,288,070 (19.5%)	926,632 (18.6%)
**2**	1,206,713 (18.3%)	989,579 (19.9%)
**3+**	2,557,751 (38.8%)	1,986,234 (40.0%)
