# Supplementary material for: Random plasma glucose predicts the diagnosis of diabetes
Source: PLoS One. 2019 Jul 19;14(7):e0219964. doi: 10.1371/journal.pone.0219964 (PMC6641200; doi:10.1371/journal.pone.0219964)
Supplement: S2 Table — (PDF) [file pone.0219964.s002.pdf]

S2 Table: Patient characteristics by number of available RPGs in baseline year

	Number of RPG measurements in baseline year			p-value (compared to ≥3)
	≥1	≥2	≥3	
**n**	1,534,064	1,410,505	942,446	
**Age (years)**	63 ± 13	63 ± 13	63 ± 12	<0.001
**Male sex (%)**	95.8%	95.9%	96.1%	<0.001
**Race (%)**				
**White**	83.8%	83.8%	82.7%	<0.001
**Black**	13.4%	13.6%	14.9%	<0.001
**Other**	2.7%	2.6%	2.4%	<0.001
**Ethnicity (%)**				
**NonHispanic/Unknown**	95.7%	95.6%	95.1%	<0.001
**Hispanic/Latino**	4.3%	4.4%	4.9%	<0.001
**BMI (kg/m^2)**	28.7 ± 5.2	28.7 ± 5.2	28.9 ± 5.4	<0.001
**Systolic BP (mmHg)**	133.9 ± 18.1	134.0 ± 18.1	134.6 ± 18.6	<0.001
**Non-HDL Chol (mg/dl)**	141.4 ± 39.0	141.2 ± 38.9	141.5 ± 39.8	<0.001
**RPG glucose (mg/dl)**	103.3 ± 15.2	103.6 ± 15.1	106 ± 16	<0.001
**Smoking (past/current) (%)**	79.9%	80.2%	81.3%	<0.001
**CVD, preexisting (%)**	33.4%	34.6%	37.5%	<0.001
**Elixhauser Index (median (Q1, Q3))**	-1 (-5, 2)	-1 (-5, 2)	-1 (-5, 3)	<0.001
**Average baseline year**	2004.6 ± 1.7	2004.5 ± 1.7	2004.4 ± 1.7	<0.001
