# Supplementary material for: Random plasma glucose predicts the diagnosis of diabetes
Source: PLoS One. 2019 Jul 19;14(7):e0219964. doi: 10.1371/journal.pone.0219964 (PMC6641200; doi:10.1371/journal.pone.0219964)
Supplement: S3 Table — (PDF) [file pone.0219964.s003.pdf]

S3 Table: Percentile distribution of median RPG levels at baseline and 1 year prior, stratified by diabetes status at end of followup period

**Percentile**	**Median RPG, 1 year before baseline to baseline year**	
	**Non-diabetes** n=847,847	**Diabetes** n=94,599
5%	83	91
10%	87	96
25%	93	103
50%	101	112
75%	108	122
90%	116	135
95%	123	145
