# Supplementary material for: Random plasma glucose predicts the diagnosis of diabetes
Source: PLoS One. 2019 Jul 19;14(7):e0219964. doi: 10.1371/journal.pone.0219964 (PMC6641200; doi:10.1371/journal.pone.0219964)
Supplement: S4 Table — (PDF) [file pone.0219964.s004.pdf]

S4 Table. ROCs and Integrated Discrimination Improvement (IDI) for prediction of diabetes at years 1, 3, and 5, stratified by sex

		Models	Year 1	Year 3	Year 5
Males	ROC AUC (95th% CI)	Model 1: Demographic factors [a]	0.703 (0.700 , 0.705)	0.702 (0.700 , 0.704)	0.701 (0.699 , 0.703)
		Model 2: Demographic + CVD factors†	0.706 (0.703 , 0.708)	0.703 (0.701 , 0.705)	0.702 (0.700 , 0.703)
		Model 3: RPG (maximum)	0.856 (0.854 , 0.858)	0.821 (0.820 , 0.823)	0.791 (0.790 , 0.793)
		Model 4: RPG (average)	0.877 (0.876 , 0.879)	0.836 (0.834 , 0.837)	0.799 (0.798 , 0.801)
		Model 5: RPG (≥2 measures at/above threshold)	0.878 (0.876 , 0.880)	0.839 (0.837 , 0.840)	0.804 (0.802 , 0.805)
		Model 6: Demographic + CVD + RPG (maximum)	0.882 (0.880 , 0.883)	0.855 (0.853 , 0.856)	0.832 (0.830 , 0.833)
		Model 7: Demographic + CVD + RPG (average)	0.899 (0.898 , 0.901)	0.869 (0.868 , 0.871)	0.843 (0.842 , 0.845)
		Model 8: Demographic + CVD + RPG (≥2 at/above threshold)	0.900 (0.898 , 0.901)	0.872 (0.870 , 0.873)	0.847 (0.846 , 0.848)
	IDI [c]	Model 1: Demographic factors [a]	reference	reference	reference
		Model 2: Demographic + CVD factors [b]	0.0007	0.0010	0.0013
		Model 3: Demographic + CVD + RPG (≥2 at/above threshold)	0.1874	0.2438	0.2616
Females	ROC AUC (95th% CI)	Model 1: Demographic factors [a]	0.746 (0.733 , 0.759)	0.735 (0.725 , 0.745)	0.730 (0.721 , 0.739)
		Model 2: Demographic + CVD factors†	0.751 (0.738 , 0.765)	0.740 (0.729 , 0.750)	0.734 (0.725 , 0.742)
		Model 3: RPG (maximum)	0.859 (0.848 , 0.869)	0.821 (0.812 , 0.830)	0.789 (0.781 , 0.798)
		Model 4: RPG (average)	0.862 (0.849 , 0.874)	0.821 (0.811 , 0.831)	0.780 (0.771 , 0.790)
		Model 5: RPG (≥2 measures at/above threshold)	0.871 (0.859 , 0.882)	0.828 (0.818 , 0.838)	0.787 (0.778 , 0.796)
		Model 6: Demographic + CVD + RPG (maximum)	0.890 (0.880 , 0.899)	0.861 (0.853 , 0.869)	0.839 (0.832 , 0.846)
		Model 7: Demographic + CVD + RPG (average)	0.898 (0.888 , 0.908)	0.868 (0.860 , 0.877)	0.842 (0.835 , 0.850)
		Model 8: Demographic + CVD + RPG (≥2 at/above threshold)	0.905 (0.896 , 0.914)	0.875 (0.867 , 0.883)	0.848 (0.840 , 0.855)
	IDI [c]	Model 1: Demographic factors [a]	reference	reference	reference
		Model 2: Demographic + CVD factors [b]	0.0022	0.0034	0.0043
		Model 3: Demographic + CVD + RPG (≥2 at/above threshold)	0.1857	0.2425	0.2611

[a] Demographic factors (age, sex, BMI, race, ethnicity)
[b] CVD factors (systolic blood pressure, non-HDL-cholesterol, smoking)
[c] $p<0.0001$ for all IDI analyses comparing prediction performances using model 1 as reference within each year
