# Supplementary material for: Random plasma glucose predicts the diagnosis of diabetes
Source: PLoS One. 2019 Jul 19;14(7):e0219964. doi: 10.1371/journal.pone.0219964 (PMC6641200; doi:10.1371/journal.pone.0219964)
Supplement: S5 Table — (PDF) [file pone.0219964.s005.pdf]

S5 Table: Sensitivity, sensitivity, specificity, and positive and negative predictive values for different cutoffs for at least 2 RPGs at/above a cutoff to predict a diagnosis of diabetes within 1, 3, and 5 years, stratified by sex

>=2 measures at/above RPG threshold (mg/dl)	n	%	Year 1				Year 3				Year 5			
			SENS [a]	SPEC [b]	PPV [c]	NPV [d]	SENS [a]	SPEC [b]	PPV [c]	NPV [d]	SENS [a]	SPEC [b]	PPV [c]	NPV [d]
**Male**														
110	340038	11.1	92.2%	64.9%	10.6%	99.5%	87.1%	66.4%	17.3%	98.5%	82.0%	67.4%	22.1%	97.1%
115	239270	7.9	86.7%	76.3%	14.2%	99.2%	78.9%	77.8%	22.3%	97.9%	71.9%	78.7%	27.5%	96.1%
120	168119	9.4	79.8%	84.2%	18.6%	98.9%	69.2%	85.5%	27.9%	97.2%	60.9%	86.2%	33.2%	95.1%
130	83233	4.4	58.9%	93.1%	27.7%	98.0%	46.6%	93.8%	37.9%	95.6%	39.0%	94.2%	42.9%	93.2%
140	42996	2.1	38.5%	96.8%	35.0%	97.2%	29.2%	97.2%	46.0%	94.4%	23.8%	97.4%	50.8%	91.9%
150	23873	2.6	25.5%	98.4%	41.9%	96.7%	19.0%	98.7%	53.9%	93.8%	15.2%	98.8%	58.5%	91.2%
**Female**														
110	9319	7.7	86.5%	76.8%	10.5%	99.4%	79.7%	78.2%	18.3%	98.4%	72.6%	79.1%	23.5%	97.0%
115	6470	5.5	80.1%	84.5%	14.1%	99.3%	70.3%	85.8%	23.2%	97.9%	61.5%	86.5%	28.7%	96.2%
120	4416	6.2	71.2%	90.0%	18.3%	99.0%	59.9%	91.0%	29.0%	97.4%	50.8%	91.5%	34.7%	95.5%
130	2113	2.8	51.5%	95.8%	27.7%	98.4%	38.0%	96.3%	38.4%	96.2%	30.3%	96.5%	43.3%	94.0%
140	1063	1.3	33.3%	98.1%	35.7%	97.9%	22.7%	98.4%	45.7%	95.4%	18.0%	98.5%	51.1%	93.1%
150	574	1.5	22.6%	99.1%	44.8%	97.6%	14.9%	99.3%	55.4%	95.0%	11.5%	99.3%	60.3%	92.7%

[a] SENS = sensitivity
[b] SPEC = specificity
[c] PPV = positive predictive value
[d] NPV = negative predictive value
