# Supplementary material for: Random plasma glucose predicts the diagnosis of diabetes
Source: PLoS One. 2019 Jul 19;14(7):e0219964. doi: 10.1371/journal.pone.0219964 (PMC6641200; doi:10.1371/journal.pone.0219964)
Supplement: S6 Table — (PDF) [file pone.0219964.s006.pdf]

S6 Table: Negative predictive values of RPG values <110 mg/dl for *not* being diagnosed with diabetes within 1, 3, and 5 years

Number of RPG values <110 mg/dl	n	%	Negative Predictive Value (NPV) for incident diabetes		
			Year 1	Year 3	Year 5
1	230,612	24.5%	98.1%	96.0%	93.8%
2	154,323	16.4%	99.5%	98.5%	97.1%
3 or more	438,766	46.6%	99.7%	99.0%	97.9%

No differences observed when stratified by sex.
