# Supplementary material for: Random plasma glucose predicts the diagnosis of diabetes
Source: PLoS One. 2019 Jul 19;14(7):e0219964. doi: 10.1371/journal.pone.0219964 (PMC6641200; doi:10.1371/journal.pone.0219964)
Supplement: S7 Table — Total n = 653,180, only subjects having 2 out of 3 glucose measurements collected at the same time of day. (PDF) [file pone.0219964.s007.pdf]

S7 Table: ROCs for prediction of diabetes by collection time of RPG

RPG collection by time of day	n	ROC (95th% CI) to predict incident diabetes		
		Year 1	Year 3	Year 5
Before 9 am	287,330	0.892 (0.888, 0.895)	0.865 (0.862, 0.868)	0.838 (0.835, 0.841)
9-11 am	211,313	0.881 (0.875, 0.886)	0.845 (0.839, 0.850)	0.815 (0.809, 0.820)
11-1 pm	80,324	0.836 (0.819, 0.853)	0.789 (0.775, 0.804)	0.756 (0.742, 0.769)
1-3 pm	42,169	0.789 (0.762, 0.816)	0.775 (0.755, 0.795)	0.740 (0.722, 0.759)
3-5 pm	16,230	0.848 (0.812, 0.883)	0.810 (0.779, 0.841)	0.756 (0.726, 0.786)
After 5 pm	15,814	0.821 (0.794, 0.849)	0.788 (0.765, 0.810)	0.759 (0.738, 0.779)

Total n=653,180, only subjects having 2 out of 3 glucose measurements collected at the same time of day.
No differences observed when stratified by sex.
