# Supplementary figures and images for: Random plasma glucose predicts the diagnosis of diabetes
Source: PLoS One. 2019 Jul 19;14(7):e0219964. doi: 10.1371/journal.pone.0219964 (PMC6641200; doi:10.1371/journal.pone.0219964)

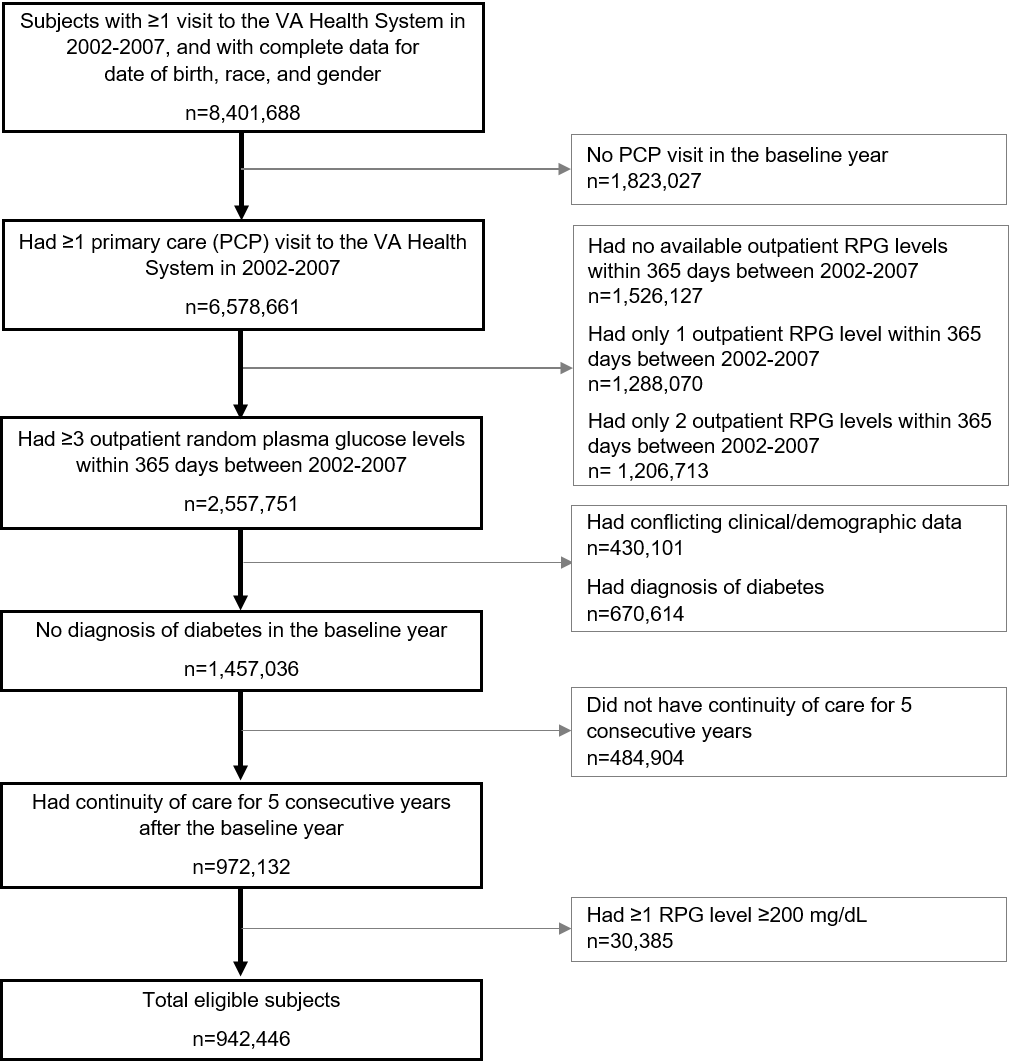

Supplement: S1 Fig — (TIF) [file pone.0219964.s008.tif]

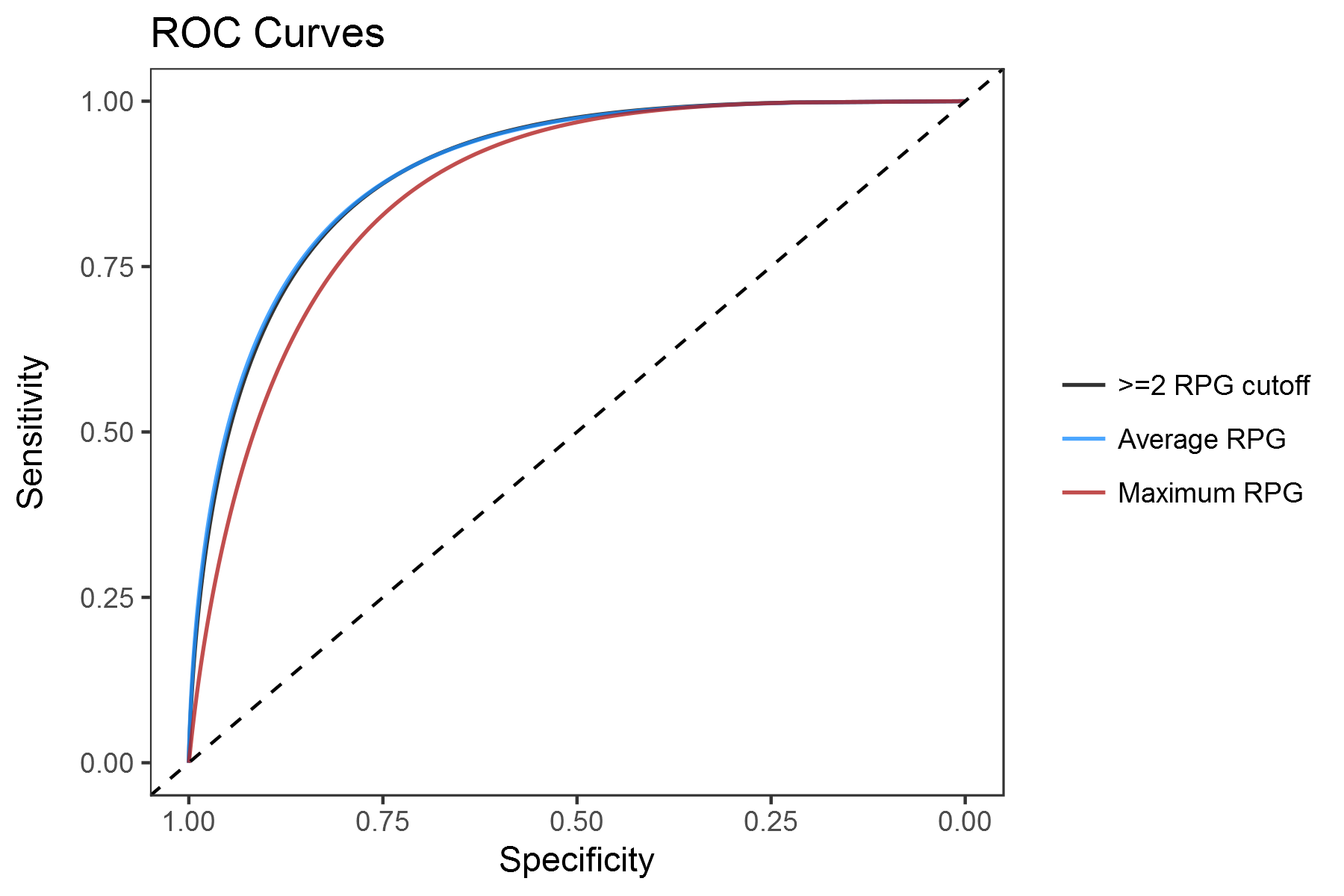

Supplement: S2 Fig — p<0.0001 for ROC AUC for average RPG vs maximum RPG, and for ≥2 RPGs at/above a cutoff vs maximum RPG; p = NS for ROC AUC for average RPG vs 2 RPGs ≥ a cutoff. (TIF) [file pone.0219964.s009.tif]
